# Advances in Palladium-Catalyzed Carboxylation Reactions

**DOI:** 10.3390/molecules27010262

**Published:** 2022-01-01

**Authors:** Lucia Veltri, Roberta Amuso, Raffaella Mancuso, Bartolo Gabriele

**Affiliations:** Laboratory of Industrial and Synthetic Organic Chemistry (LISOC), Department of Chemistry and Chemical Technologies, University of Calabria, Via Pietro Bucci 12/C, 87036 Arcavacata di Rende, Italy; robyamuso@gmail.com (R.A.); raffaella.mancuso@unical.it (R.M.)

**Keywords:** carbon dioxide, carboxylation, catalysis, heterocycles, palladium

## Abstract

In this short review, we highlight the advancements in the field of palladium-catalyzed carbon dioxide utilization for the synthesis of high value added organic molecules. The review is structured on the basis of the kind of substrate undergoing the Pd-catalyzed carboxylation process. Accordingly, after the introductory section, the main sections of the review will illustrate Pd-catalyzed carboxylation of olefinic substrates, acetylenic substrates, and other substrates (aryl halides and triflates).

## 1. Introduction

The efficient incorporation of carbon dioxide into an organic substrate (carboxylation) under catalytic conditions to give high value added molecules is one of the most important and fascinating areas of current organic synthesis. In fact, carbon dioxide is a nonflammable, inexpensive and largely available C-1 feedstock. Moreover, the efficient conversion of CO_2_ into organic compounds is a very attractive synthetic approach. In fact, it allows converting an important waste (it is well known that carbon dioxide is produced in enormous amounts from the combustion of fossil fuels for the production of energy) into a variety of useful compounds, which can find application as fuels or in the pharmaceutical or material fields. Accordingly, many efforts have been devoted by the scientific community to develop novel efficient and sustainable carboxylation methods, in particular under catalytic conditions, during the last years [1,2,3,4,5,6,7,8,9,10,11,12,13,14,15,16,17,18,19,20,21,22,23,24,25,26,27,28,29,30,31,32,33,34].

This short review is intended to present paradigmatic examples of carboxylation processes based on palladium catalysis, with particular emphasis to the more recently reported methods (coverage: from 1980ies to date). Only processes in which carbon dioxide is fully incorporated into the organic substrates will be considered, while the reactions in which CO_2_ is incorporated as a carbonyl function only, resulting in indirect carbonylation rather than carboxylation, are beyond the scope of this review.

## 2. Palladium-Catalyzed Incorporation of Carbon Dioxide into Olefinic Substrates

**Scheme 1:**
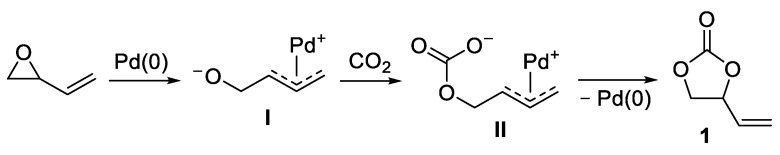
Pd(0)-catalyzed carboxylation of 2-vinyloxirane to 4-vinyl-1,3-dioxolan-2-one **1**.

**Scheme 2:**
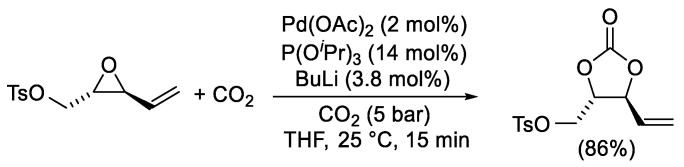
Synthesis of *trans*-(2-oxo-5-vinyl-1,3-dioxolan-4-yl)methyl 4-methylbenzenesulfonate.

Suitably functionalized olefins are excellent substrates for the Pd-catalyzed incorporation of carbon dioxide to give high value added heterocyclic compounds. For example, vinyl epoxides undergo a Pd(0)-promoted ring-opening process with formation of a π-allylpalladium alkoxide intermediate **I**, which can attack CO_2_ to give a π-allylpalladium carbonate **II** that then undergoes intramolecular nucleophilic attack to give vinyl-substituted 5-membered cyclic carbonates **1**, as shown in Scheme 1. This kind of process was independently disclosed by the groups of Fujinami [35] and Trost [36,37] in the 1980s; an example of synthetic application is shown in Scheme 2 (in this and in all the following schemes of the review, unreactive ligands on palladium are not shown for clarity) [38].

**Scheme 3:**
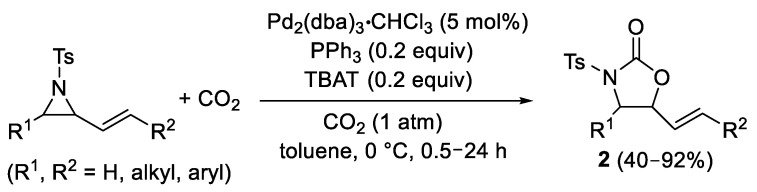
Synthesis of 5-vinlyloxazolidinones **2** from vinylaziridines.

In a similar manner, 5-vinyloxazolidinones **2** can be synthesized from vinylaziridines. For example, using a catalytic system consisting of Pd_2_(dba)_3_/PPh_3_/TBAT (TBAT = tetrabutylammonium difluorotriphenylsilicate), in toluene as the solvent and under particularly mild reaction conditions (0 °C and atmospheric pressure of CO_2_), a variety of 5-vinyloxazolidinones was prepared (Scheme 3). The process showed high regioselectivity and was also diastereospecific [39].

**Scheme 4:**
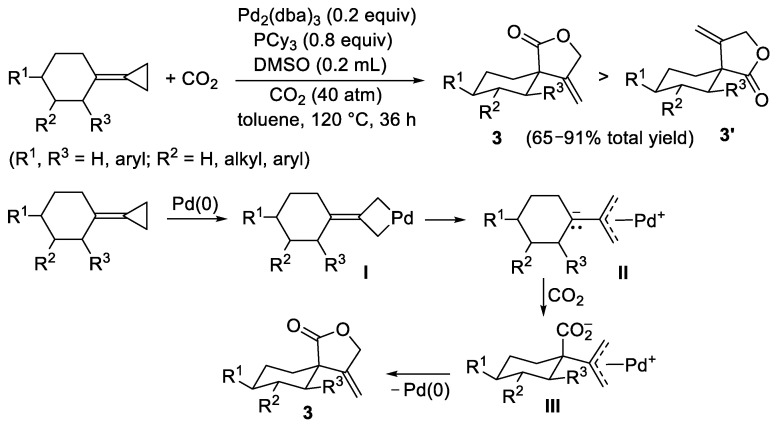
Synthesis of five-membered lactones **3** and **3′** from cycloalkylidenecyclopropanes.

Cycloalkylidenecyclopropanes, bearing the highly reactive cyclopropane ring, have been reported to undergo ring-opening carboxylation under relatively mild conditions to yield five-membered lactones, as shown in Scheme 4 for the formation of **3** and **3′** [40]. Reactions were carried out in toluene at 120 °C under 40 atm of CO_2_, in the presence of Pd_2_(dba)_3_/PCy_3_ as the catalytic system and dimethyl sulfoxide (DMSO) as additive. The reaction showed a certain degree of diastereoselectivity, favoring the formation of the diastereoisomer with the carbonyl group in the axial position **3** in most cases. The proposed mechanism involves the initial oxidative insertion of Pd(0) into the cyclopropane ring with formation of a four-membered Pd(II) palladacycle intermediate **I**, as shown in Scheme 4, which may undergo a ring opening process to give a zwitterionic π-allypalladium complex **II**. The latter inserts CO_2_ by carbanion attack to give a carboxylate zwitterionic intermediate **III** (only the intermediate leading to the major diastereoisomer is shown in the scheme), from which the final product **3** is formed by intramolecular attack of the carboxylate group to the π-allyl moiety, with regeneration of the Pd(0) catalyst (Scheme 4).

**Scheme 5:**
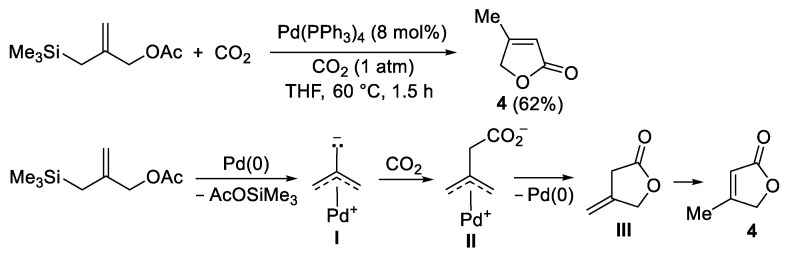
Synthesis of 4-methylfuran-2(5*H*)-one **4** from 2-((trimethylsilyl)methyl)allyl acetate.

Functionalized allylic substrates can also undergo carbon dioxide fixation under Pd(0) catalysis. As an example, 2-(acetoxymethyl)-3-(trimethylsilyl)propenes were converted into 2(5*H*)-furanones when allowed to react with CO_2_ (1 atm) in 1,2-dimethoxyethane (DME) or THF at 60–75 °C in the presence of Pd(PPh_3_)_4_, although in modest to moderate isolated yields (35–62%), as exemplified in Scheme 5 for the formation of **4** [41]. Mechanistically, the reaction follows a pathway similar to that seen in Scheme 1, as the key intermediate is a zwitterionic π-allylpalladium complex **I** [formed by oxidative addition of the substrate to Pd(0)], which reacts with CO_2_ to give a zwitterionic carboxylate complex **II**. The latter undergoes cyclization (by intramolecular nucleophilic attack of the carboxylate to the π-allylpalladium system), with regeneration of Pd(0) and formation of a 4-methylenedihydrofuran-2(3*H*)-one intermediate **III**, which eventually isomerizes to the final 2(5*H*)-furanone product **4** (Scheme 5).

**Scheme 6:**
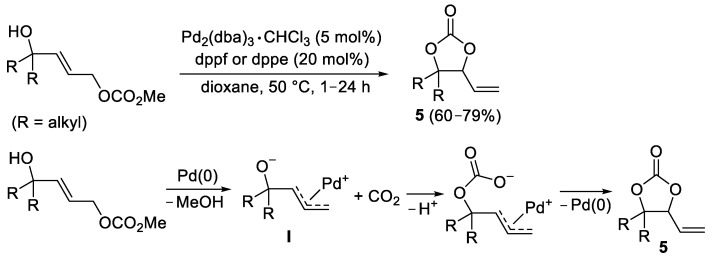
Synthesis of 4-vinyl-1,3-dioxolan-2-ones **5** from (*E*)-4-hydroxybut-2-en-1-yl methyl carbonates.

Vinyl-substituted 5-membered cyclic carbonates **5** were synthesized from allylic carbonates by a sequential CO_2_ elimination–fixation process, as shown in Scheme 6, with formal CO_2_ recycling, again through the formation of a zwitterionic π-allylpalladium complex **I** [42]. Reactions were performed in the presence of Pd_2_(dba)_3_ as catalyst in the presence of dppf [1,1′-bis(diphenylphosphino)ferrocene] or dppe [1,2-bis(diphenylphosphino)ethane] as ligand, in dioxane at 50 °C under inert atmosphere. The process was shown to be enantiospecific when starting from nonracemic allylic carbonates to yield nonracemic cyclic carbonates.

**Scheme 7:**
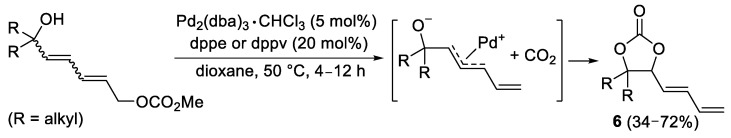
Synthesis of dienylic 5-membered cyclic carbonates **6** from 6-methoxycarbonyloxy-2,4-hexadien-1-ols.

In a subsequent work, the same research group reported the synthesis of dienylic 5-membered cyclic carbonates **6** from 6-methoxycarbonyloxy-2,4-hexadien-1-ols under similar conditions [Pd_2_(dba)_3_ as catalyst in the presence of dppe or dppv (1,2-bis(diphenylphosphino)ethylene) as ligand, in dioxane at 50 °C], as shown in Scheme 7 [43].

**Scheme 8:**
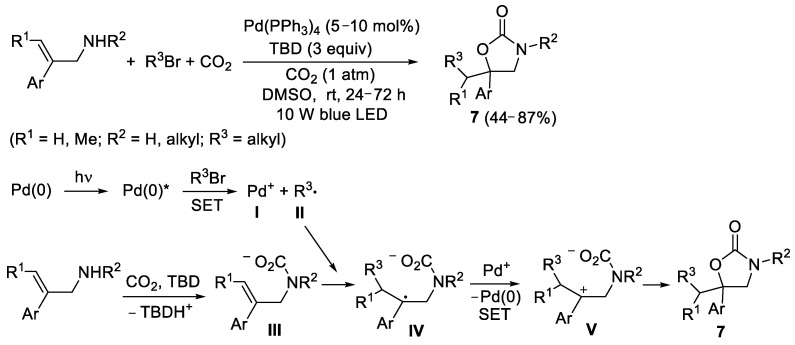
Synthesis of 2-oxazolidinones **7** from allylamines and alkyl bromides.

Allylamines have been reported to react with alkyl bromides and carbon dioxide (1 atm) in DMSO at room temperature, in the presence of Pd(PPh_3_)_4_ as catalyst and 1,5,7-triazabicyclo[4.4.0]dec-5-ene (TBD) as base and under visible light irradiation (10 W blue LED lamp), to give 2-oxazolidinones **7** in good yields (Scheme 8) [44]. Experimental data were in agreement with a radical mechanism, which starts with the photoexcitation of Pd(0) followed by single electron transfer (SET) with the alkyl bromide to give a Pd(I) species **I** and an alkyl radical **II**. The latter reacts with the double bond of the carbamate intermediate **III** formed by the attack of the amino group of the allylamine substrate to CO_2_ in the presence of TBD, thus leading to a radical anion species **IV**. This species undergoes another SET process with Pd(I) with regeneration of the Pd(0) catalyst and formation of a zwitterionic intermediate **V**, whose cyclization by intramolecular nucleophilic attack finally affords the oxazolidinone product **7** (Scheme 8).

**Scheme 9:**
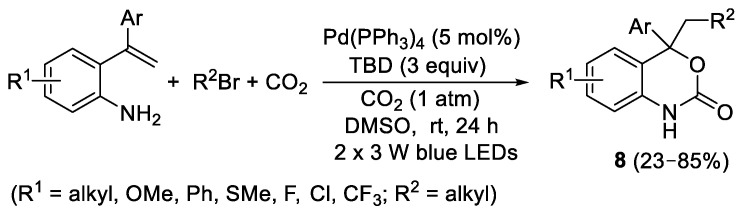
Synthesis of 1,4-dihydro-2*H*-3,1-benzoxazin-2-ones **8** from 2-(1-arylvinyl)anilines and alkyl bromides.

In a very similar manner, 1,4-dihydro-2*H*-3,1-benzoxazin-2-ones **8** were recently synthesized from 2-(1-arylvinyl)anilines, alkyl bromides, and carbon dioxide in the presence of the same catalyst [Pd(PPh_3_)_4_] and base (TBD) and under visible light irradiation (2 × 3 W blue LED lamps), at room temperature and under atmospheric pressure of CO_2_ (Scheme 9) [45].

**Scheme 10:**
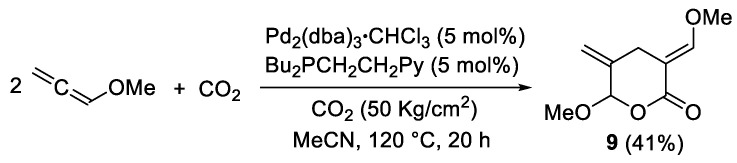
Synthesis of (*E*)-6-methoxy-3-(methoxymethylene)-5-methylenetetrahydro-2*H*-pyran-2-one **9** from methoxyallene.

Functionalized allenes are also useful substrate for Pd-catalyzed CO_2_ incorporation. As early as 1992, Tsuda and coworkers reported the dimerizative carboxylation of methoxyallene into (*E*)-6-methoxy-3-(methoxymethylene)-5-methylenetetrahydro-2*H*-pyran-2-one **9** (41% isolated yield), catalyzed by Pd_2_(dba)_3_ in the presence of Bu_2_PCH_2_CH_2_Py (Py = pyridyl) in MeCN as the solvent at 120 °C and under 50 kg/cm^2^ pressure of carbon dioxide (Scheme 10) [46]. The methoxy substituent was essential to the success of the reaction. A palladacycle intermediate was proposed to be the key intermediate in product formation.

**Scheme 11:**
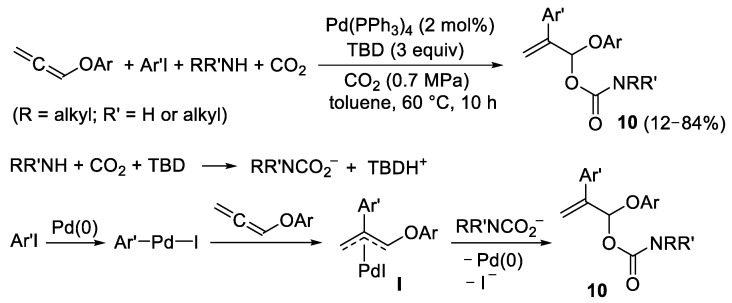
Synthesis of 1-aryloxy-2-arylallyl carbamates **10** from aryloxyallenes, aryl iodides, and amines.

More recently, aryloxyallenes have been reported to undergo a multicomponent reaction with amines, carbon dioxide, and aryl iodides, catalyzed by Pd(PPh_3_)_4_ in the presence of TBD, to give 1-aryloxy-2-arylallyl carbamates **10**, according to Scheme 11 [47]. From a mechanistic point of view, the process is believed to begin with the oxidative addition of the aryl iodide to Pd(0) to give an ArPd(II) complex, which then inserts the allenyl moiety of the substrate thus leading to a π-allylpalladium complex **I**. The final product is finally obtained by regioselective nucleophilic attack to the π-allylpalladium moiety by the carbamate formed by the reaction between the amine and CO_2_ in the presence of TBD (Scheme 11).

**Scheme 12:**
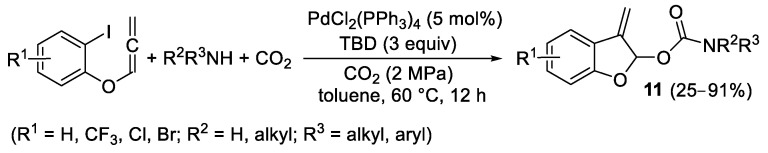
Synthesis of 3-methylene-2,3-dihydrobenzofuran-2-yl carbamates **11** from 1-iodo-2-(propa-1,2-dien-1-yloxy)benzenes and amines.

When the aryloxy and Ar–I functional groups were present in the same substrate in relative *ortho* positions, 3-methylene-2,3-dihydrobenzofuran-2-yl carbamates **11** were obtained by a similar sequence of mechanistic steps, the carbamate nucleophilic attack to the π-allyl system occurring in this case intramolecularly (Scheme 12) [48]. Interestingly, (1-tosyl-1*H*-indol-3-yl)methyl carbamates were selectively formed when starting from 2-iodo-*N*-(propa-1,2-dien-1-yl)-*N*-tosylanilines, by an inversion of regiochemistry in the intramolecular nucleophilic attack, probably owing to the steric hindrance exerted by the tosyl group on nitrogen.

**Scheme 13:**
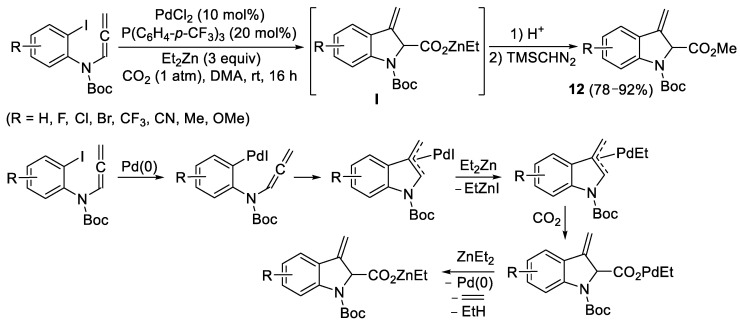
Synthesis of methyl 1-Boc-3-methyleneindoline-2-carboxylates **12** by from *N*-Boc-2-iodo-*N*-(propa-1,2-dien-1-yl)anilines.

In a related work, *N*-Boc-2-iodo-*N*-(propa-1,2-dien-1-yl)anilines (Boc = *tert*-butyloxycarbonyl) were allowed to react with ZnEt_2_ and CO_2_ (1 atm) and room temperature in the presence of PdCl_2_ as the palladium source and P(C_6_H_4_-*p*-CF_3_)_3_ as ligand to give a ((1-Boc-3-methyleneindoline-2-carbonyl)oxy)(ethyl)zinc intermediate **I**. This was transformed into methyl 1-Boc-3-methyleneindoline-2-carboxylates **12** by acidic quenching and subsequent reaction with TMSCHN_2_ (TMS = trimethylsilyl) (Scheme 13) [49].

**Scheme 14:**
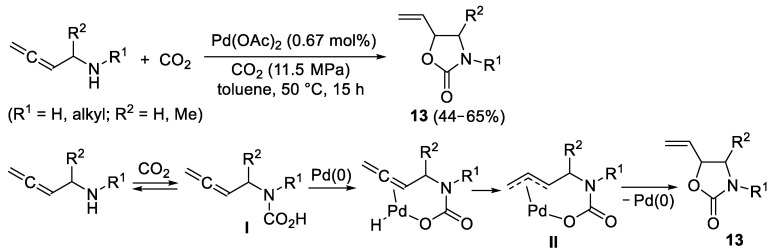
Synthesis of 5-vinyl-2-oxazolidinones **13** from α-allenyl amines.

Ikayara and coworkers reported the reaction of α-allenyl amines with dense CO_2_ (11.5 MPa) to give 5-vinyl-2-oxazolidinones **13** under the catalysis of Pd(0), obtained in situ from palladium acetate, as shown in Scheme 14 [50]. The proposed mechanism starts with the formation of a carbamate intermediate **I** (from the reaction between the substrate and CO_2_) followed by oxidative addition of the –OH group to Pd(0). Insertion of the internal allenyl double bond into the ensuing Pd–H bond then takes place, with formation of a π-allylpalladium complex **II**. Cyclization with reductive elimination of Pd(0) finally yields the oxazolidinone product **13** (Scheme 14).

**Scheme 15:**
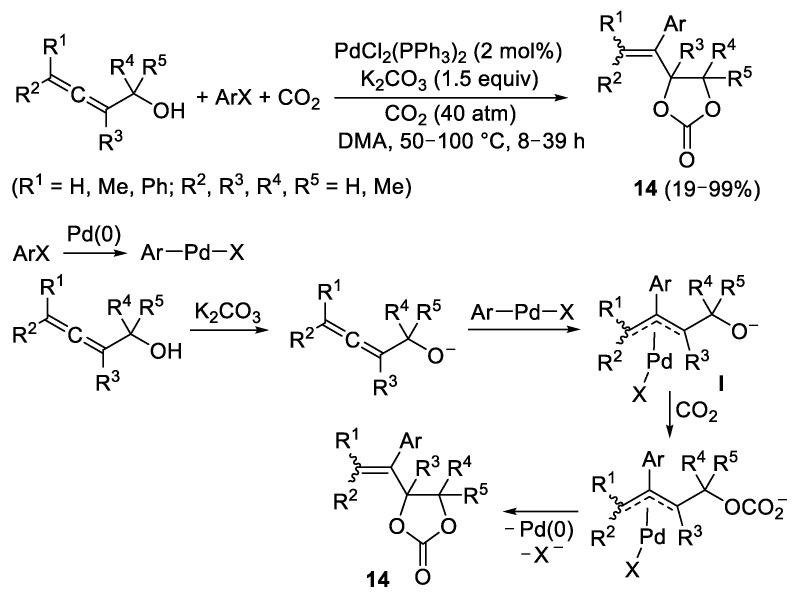
Synthesis of 4-vinyl-1,3-dioxolan-2-ones **14** from 2,3-dienols and aryl halides.

In 1999, Inoue and coworkers reported the Pd(0)-catalyzed formation of vinyl-substituted cyclic carbonates from 2,3-dienols or 3,4-dienols, aryl or vinyl halides, and CO_2_, as exemplified in Scheme 15 for the synthesis of 4-vinyl-1,3-dioxolan-2-ones **14** from 2,3-dienols and aryl halides [51]. The process, carried out in DMA (*N*,*N*-dimethylacetamide) at 50–100 °C under 40 atm of CO_2_, in the presence of Pd(PPh_3_)_4_ as catalyst and K_2_CO_3_ as base, took place through oxidative addition of the halide to Pd(0), followed by insertion of the allenyl moiety of the deprotonated substrate to give a π-allylpalladium complex **I**. The final product **14** was then formed by attack of the anionic oxygen to CO_2_ followed by intramolecular nucleophilic attack of the ensuing carbonate moiety to the π-allylpalladium system, with regeneration of Pd(0) (Scheme 15).

**Scheme 16:**
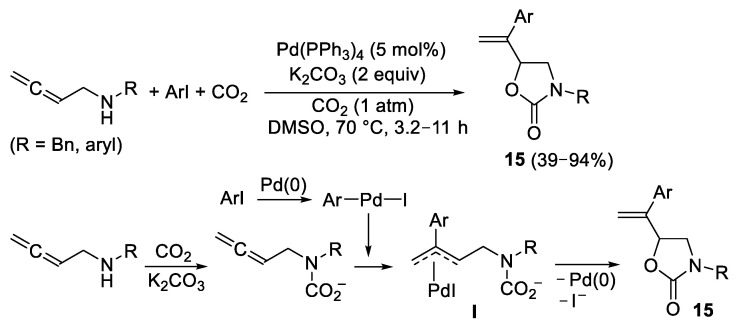
Synthesis of 5-vinyl-2-oxazolidinones **15** from 2,3-allenyl amines and aryl iodides.

In a similar way, 5-vinyl-2-oxazolidinones **15** were synthesized from 2,3-allenyl amines, aryl iodides and CO_2_ (1 atm) in the presence of Pd(PPh_3_)_4_ and K_2_CO_3_ as the base, in DMSO at 70 °C (Scheme 16) [52]. The reaction starts with the oxidative addition of the aryl iodide to Pd(0) to give an Ar–Pd–I complex, which inserts the allenyl moiety of the π-allylpalladium carbamate intermediate **I** formed by the reaction between the allenyl amine and CO_2_. Intramolecular nucleophilic attack of the carbamate to the π-allylpalladium moiety eventually leads to the vinyloxazolidinone product **15** with regeneration of Pd(0) (Scheme 16). Interestingly, the use of the ligand Gorlos-Phos•HBF_4_ (Gorlos-Phos = dicyclohexyl(2,6-diisopropoxyphenyl)phosphane) allowed a stereoselective synthesis of (*Z*)-5-alkenyloxazolidin-2-ones when starting from 4-monosubstituted 2,3-allenyl amines [53].

**Scheme 17:**
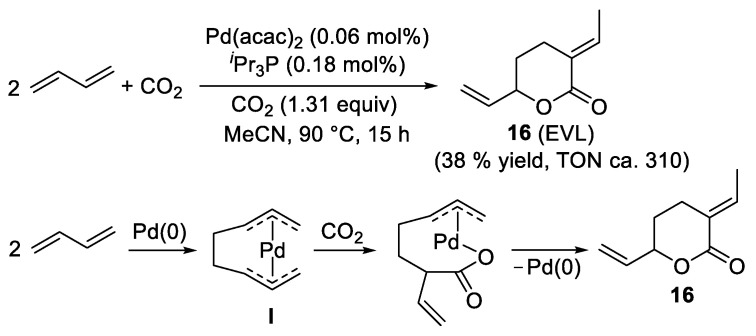
Synthesis of 2-ethylidene-6-hepten-5-olide **16** (EVL) from 1,3-butadiene.

Conjugate dienes are also reactive toward carbon dioxide under palladium catalysis. Following the pioneering studies performed by the groups of Inoue [54,55] and Musco [56,57], in 1983 Behr and coworkers reported the reaction of 1,3-butadiene with CO_2_ carried out in the presence of Pd(acac)_2_ as the palladium source (0.18%) and *^i^*Pr_3_P as ligand, in acetonitrile at 90 °C, to give 3-ethylidene-6-vinyltetrahydro-2*H*-pyran-2-one (or 2-ethylidene-6-hepten-5-olide, EVL) **16** in 38% isolated yield and a TON (Turnover Number) of ca. 310 mol of product per mol of palladium used (Scheme 17) [58,59]. Interestingly, this 6-membered lactone could be isomerized into the corresponding 5-membered one [3-ethyl-5-propylidenefuran-2(5*H*)-one, 17% GLC yield] under the same reaction conditions, but with a higher catalyst loading (1.19%) [59]. EVL is a useful precursor for the preparation of different high value added products [60,61,62,63,64], including polymers [62,63,64], such as high molecular weight polymers with a carbon dioxide content of 33 mol%, obtained by the δ-lactone free-radical polymerization [64]. Mechanistically, the telomerization process leading to EVL was believed to occur through the formation of a bis-π-allylpalladium complex **I** [from the reaction between butadiene and Pd(0)], followed by CO_2_ insertion and cyclization with reductive elimination of Pd(0) (Scheme 17).

The process was later studied by Dinjus and Leitner [65], among others [66,67,68,69], who were able for the first time to isolate and characterize by NMR the mixture of isomeric lactones [6-(prop-1-en-2-yl)-3-(propan-2-ylidene)tetrahydro-2*H*-pyran-2-one and 6-methyl-3-(propan-2-ylidene)-6-vinyltetrahydro-2*H*-pyran-2-one] obtained from isoprene [65]. These studies evidenced the importance of the use of a hindered phosphine ligand as well as of a nitrile solvent for the success of the reaction.

More recently, various catalytic systems have been developed to perform the telomerization of butadiene with CO_2_ to give lactones, including Pd(OAc)_2_ in the presence of ferrocenylphosphine ligands, such as 1,1′-bis(diisopropylphosphino)ferrocene (disoppf) [70], Pd(acac)_2_/PCy_3_ [62,71], Pd(acac)_2_/PPh_3_ [64], Pd_2_(dba)_3_/4-(2-(diphenylphosphino)phenyl)morpholine [72], Pd(acac)_2_/TBAAc (TBAAc = tetrabutylammonium acetate) [63,73], Pd(dba)_2_/TOMPP [TOMPP = tris-(*o*-methoxyphenyl)phosphine] [74], and Pd(OAc)_2_/TPMPP/H_2_Q/DIPEA [TPMPP = tris-(*p*-methoxyphenyl)phosphine, H_2_Q = *p*-hydroquinone, DIPEA = *N*,*N*-diisopropylethylamine] [75]. In particular, using the last catalytic system, EVL was obtained in 96% selectivity and with an unprecedented TON of ca. 4500, by performing the reaction in MeCN at 70 °C for 5 h [75].

## 3. Palladium-Catalyzed Incorporation of Carbon Dioxide into Acetylenic Substrates

Acetylenic substrates have been reported to undergo several important carboxylation processes catalyzed by palladium, with formation of high value added compounds.

**Scheme 18:**
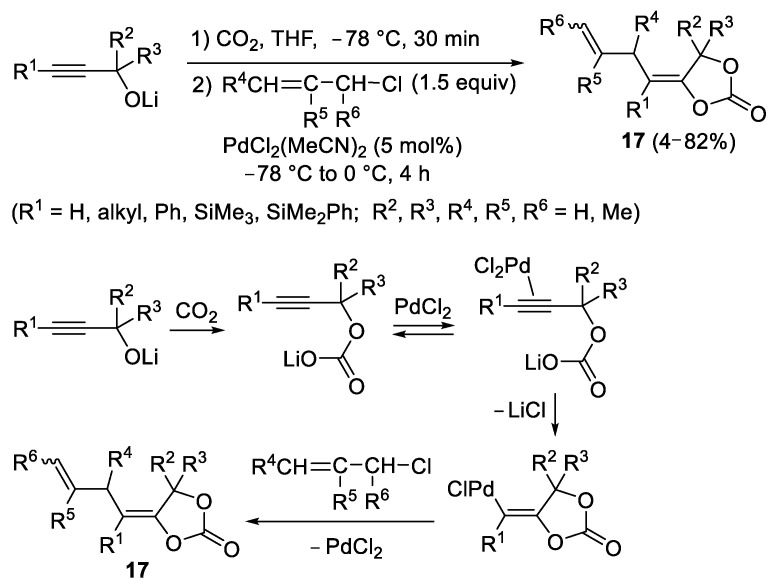
Synthesis of 4-(but-3-en-1-ylidene)-1,3-dioxolan-2-ones **17** from lithium 2-alkyn-1-olates.

In 1986, Utimoto and coworkers reported the Pd(II)-catalyzed reaction of lithium 2-alkynyl carbonates (obtained from the reaction between lithium 2-alkyn-1-olates with CO_2_) with allylic chlorides to give 4-(but-3-en-1-ylidene)-1,3-dioxolan-2-ones **17** (Scheme 18) [76]. The synthetic transformation was carried out in a one-pot, two-step fashion, by allowing ynolate to react with CO_2_ first (in THF at −78 °C) and then adding the allyl chloride together with the catalyst PdCl_2_(MeCN)_2_ at the same temperature followed by stirring at 0 °C for 4 h, as shown in Scheme 18.

**Scheme 19:**
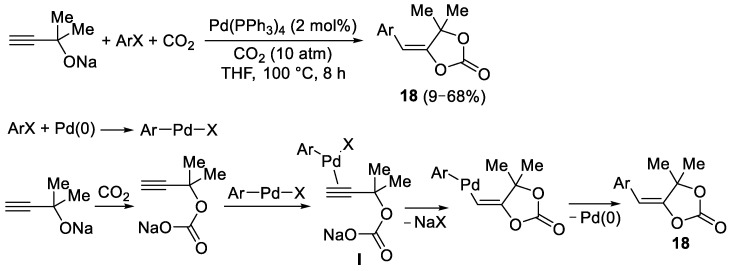
Synthesis of (*E*)-4-(arylmethylene)-1,3-dioxolan-2-ones **18** from sodium 2-alkyn-1-olates and aryl halides.

Later on, Inoue and coworkers reported the Pd(0)-catalyzed reaction between sodium 2-alkyn-1-olates bearing a terminal triple bond, aryl halides, and CO_2_ to give cyclic carbonates **18** [(*E*)-4-(arylmethylene)-1,3-dioxolan-2-ones], as shown in Scheme 19 [77]. Reactions were carried out under 10 atm of CO_2_, in THF at 100 °C and in the presence of 2 mol% of Pd(PPh_3_)_4_. In this process, the initial oxidative addition of the aryl halide to Pd(0) leads to an Ar–Pd–X complex, which electrophilically activates the triple bond toward the *anti* 5-*exo*-*dig* intramolecular nucleophilic attack by the carbonate anion **I** formed by the reaction between the ynolate and CO_2_ (Scheme 19).

**Scheme 20:**
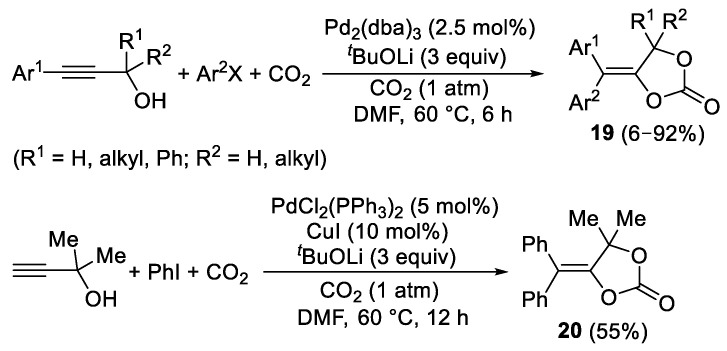
Synthesis of 5-(diarylmethylene)-1,3-dioxolan-2-ones **19** and **20** from propargyl alcohols and aryl halides.

2-Ynolates can also be formed in situ starting by deprotonation of propargyl alcohols in the presence of a suitable base. For example, recently a variety of propargyl alcohols with the triple bond substituted with an aryl group were converted into 5-(diarylmethylene)-1,3-dioxolan-2-ones **19** by their Pd(0)-catalyzed reaction with aryl halides and CO_2_ (1 atm), carried out in the presence of Pd_2_(dba)_3_ as catalyst and *^t^*BuOLi as the base (Scheme 20) [78]. Interestingly, with substrates bearing a terminal triple bond, a sequential Sonogashira coupling–carboxylation process took place, as exemplified in Scheme 20 for the case of the reaction of 2-methylbut-3-yn-2-ol with PhI (3 equiv) and CO_2_. The reaction, carried out in the presence of 5 mol% of PdCl_2_(PPh_3_)_2_ as the catalyst precursor, CuI (10 mol%) as cocatalyst, and *^t^*BuOLi as base (3 equiv), led to formation of 5-(diphenylmethylene)-4,4-dimethyl-1,3-dioxolan-2-one **20** in 55% yield (Scheme 20).

**Scheme 21:**
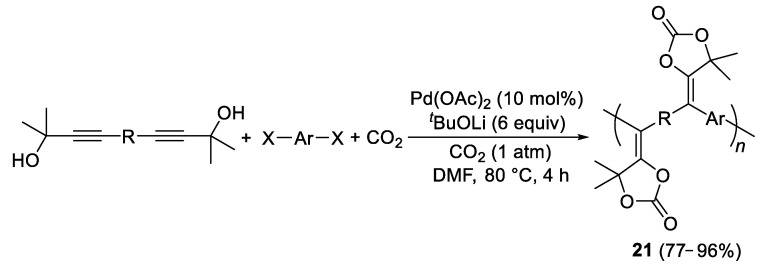
Formation of five-membered cyclic carbonate-based polymers **21** from bis(propargylic alcohol)s and aryl dihalides.

Polymeric materials can be obtained starting from bis(propargylic alcohol)s and aryl dihalides. Thus, linear and hyperbranched five-membered cyclic carbonate-based polymers **21** with high weight-average molecular weights (up to 42,500, 96% yield) were recently produced by allowing to react bis(propargylic alcohol) monomers and aryl dihalide monomers with CO_2_ (1 atm) in DMF in the presence of Pd(OAc)_2_ as catalyst precursor and *^t^*BuOLi as the base (Scheme 21) [79].

**Scheme 22:**
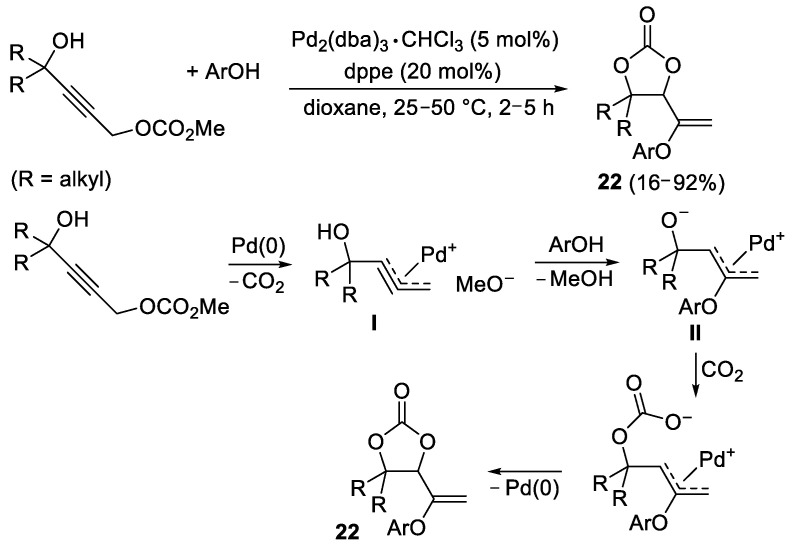
Synthesis of 4-(1-aryloxyvinyl)-1,3-dioxolan-2-ones **22** from 4-hydroxy-2-yn-1-yl methyl carbonates and phenols.

With propargylic substrates bearing a potential leaving group, Pd-catalyzed CO_2_ sequential elimination–fixation may occur through the formation of π-propargylpalladium species, in a similar way as seen in Section 2 for allylic carbonates (Scheme 6 and Scheme 7) [80]. Thus, in 2001, Yoshida and Hihara reported the synthesis of aryloxyvinyl-substituted 5-membered cyclic carbonates **22** by the reaction of 4-hydroxy-2-yn-1-yl methyl carbonates with phenols, carried out in the presence of Pd_2_(dba)_3_ and dppe in dioxane at 25–50 °C (Scheme 22) [81]. Higher product yields were obtained by performing the reaction under 1 atm of CO_2_ [81,82,83]. The reaction was also shown to be enantioselective (*ee*s up to 93%) in the presence of the nonracemic ligand (*S*)-BINAP [82] and enantiospecific (with chirality transfer) when starting from nonracemic substrates [83]. The process begins with the reaction between the substrate and Pd(0) to give carbon dioxide and π-propargylpalladium methoxide complex **I**. The latter then undergoes nucleophilic attack by phenol leading to a π-allylpalladium intermediate **II**, from which the final product is formed by attack to CO_2_ followed by intramolecular nucleophilic attack of the ensuing carbonate to the π-allylpalladium moiety (Scheme 22).

**Scheme 23:**
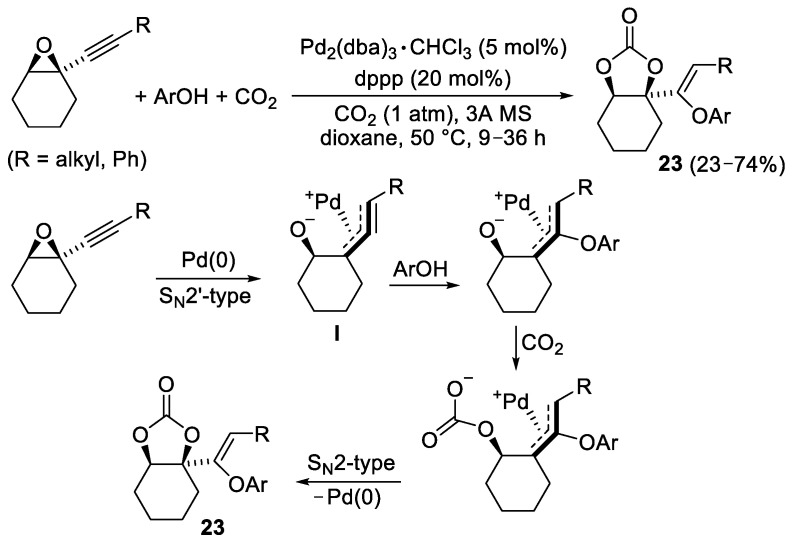
Synthesis of 2-substituted (1-aryloxyvinyl)hexahydrobenzo[*d*][1,3]dioxol-2-ones **23** from *tran*s-1-ethynyl-7-oxabicyclo[4.1.0]heptanes and phenols.

More recently, some propargylic epoxides with internal triple bond (*tran*s-1-ethynyl-7-oxabicyclo[4.1.0]heptanes, in particular) were also used as substrates under similar conditions, with the addition of 3A molecular sieves (MS) (presumably to avoid substrate ring opening by water) and under 1 atm of CO_2_ (Scheme 23) [84]. The reaction, leading to 2-substituted (1-aryloxyvinyl)hexahydrobenzo[*d*][1,3]dioxol-2-ones **23** in modest to good yields, took place through the same kind of mechanistic route seen above for propargylic carbonates (Scheme 22), the key π-propargylpalladium zwitterionic complex **I** being this time formed by Pd(0)-promoted epoxide ring opening through an *anti* S_N_2’-type attack (Scheme 23).

**Scheme 24:**
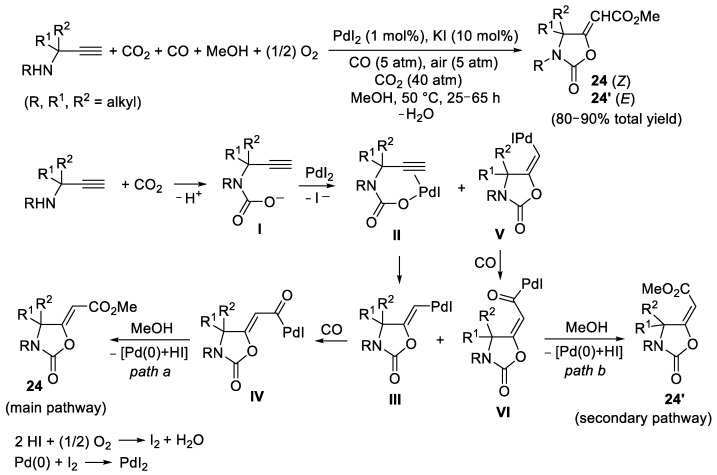
Synthesis of 5-(methoxycarbonylmethylene)oxazolidin-2-ones **24** and **24′** from propargyl amines.

Propargyl amines are excellent substrates for the Pd-catalyzed CO_2_ incorporation to give high value added oxazolidinone derivatives. In 1997, our research group reported the first example of catalytic sequential incorporation of both carbon oxides (carbon dioxide and carbon monoxide) into an organic substrate, α,α’-disubstutited propargylamines in particular [85]. The process was catalyzed by Pd(II) and took place in the presence of PdI_2_ (1 mol%), KI, MeOH (also used as solvent) at 50 °C for 25–65 h, under 50 atm of a 8:1:1 mixture of CO_2_-CO-air, to give mixtures of 5-(methoxycarbonylmethylene)oxazolidin-2-ones **24** (with *Z* configuration of the double bond) and **24**′ (with *E* configuration around the exocyclic double bond) in 80–90% total yields (*Z*/*E* ratio from 2:1 to 3:1) (Scheme 24) [85,86]. Overall, the reaction corresponded to an oxidative carbonylation [87,88,89,90,91,92,93,94,95,96,97,98,99] of the carbamate species initially formed by nitrogen attack to CO_2_, with oxygen (from air) as the external oxidant and with formation of water as benign coproduct. More specifically, the anionic carbamate **I** (formed from the reaction between the propargylamine substrate and CO_2_) led to the main product, (*Z*)-5-(methoxycarbonylmethylene)oxazolidin-2-one **24**, through the formation of a palladium carbamate complex **II**, followed by intramolecular *syn* 5-*exo*-*dig* insertion of the triple bond to give **III**, CO insertion to **IV**, and nucleophilic displacement by MeOH (Scheme 24, *path a*) [85,86]. On the other hand, intermediate **I** could also undergo *anti* 5-*exo*-*dig* nucleophilic attack of the anionic carbamate moiety to the triple bond coordinated to Pd(II), leading to an *E*-vinylpalladium complex **V**. Carbon monoxide insertion then took place, with formation of an *E*-acylpalladium intermediate **VI**, from which the final (*E*)-5-(methoxycarbonylmethylene)oxazolidin-2-one **24**′ was formed by nucleophilic displacement by MeOH (Scheme 24, *path b*). In either case, Pd(0) was formed together with the organic products. The overall process became catalytic thanks to a very efficient reoxidation of Pd(0) to Pd(II), involving the initial oxidation of 2 mol of HI (also formed during the reaction) by oxygen (from air) to produce iodine, followed by the oxidative addition of I_2_ to Pd(0) to give back PdI_2_ (Scheme 24) [100,101].

This sequential carboxylation–oxidative alkoxycarbonylation of propargyl amines to 5-(methoxycarbonylmethylene)oxazolidin-2-ones still today represents the only example reported in the literature of Pd(II)-catalyzed incorporation of both CO_2_ and CO into an organic substrate.

**Scheme 25:**
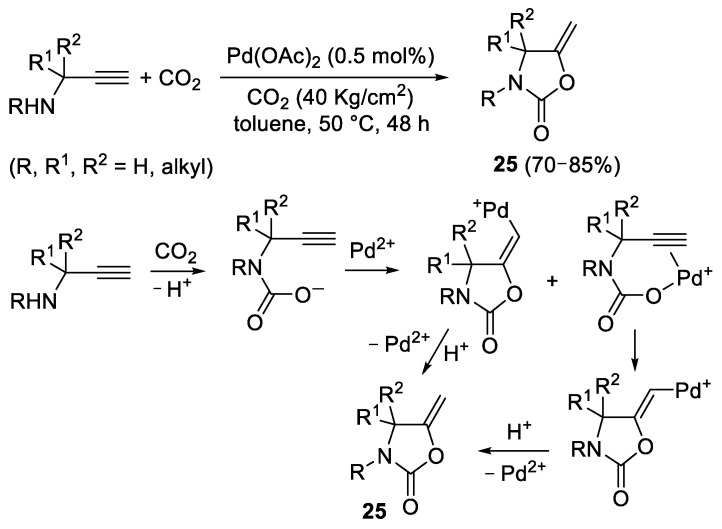
Synthesis of 5-methyleneoxazolidin-2-ones **25** from propargyl amines.

The Pd-catalyzed incorporation of CO_2_ alone (without CO) into propargylic amines has also been reported. Thus, in 2002, Shi and Shen published the Pd(II)-catalyzed carboxylation of these substrates under 40 Kg/cm^2^ of CO_2_ to give methyleneoxazolidinones **25**, using Pd(OAc)_2_ as catalyst precursor, in toluene as the solvent at 50 °C for 48 h (Scheme 25) [102]. Products were possibly formed through mechanistic pathways similar to those seen before in Scheme 24, as shown in Scheme 25.

**Scheme 26:**
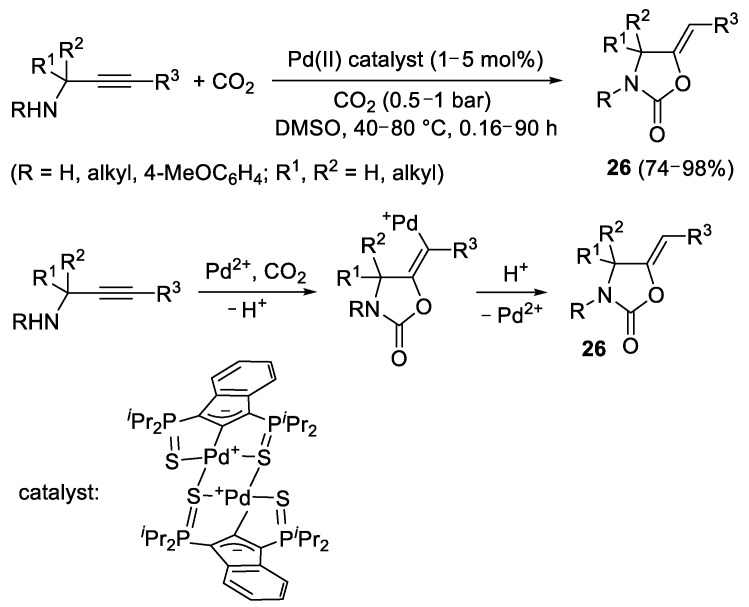
Synthesis of 5-alkylideneoxazolidin-2-ones **26** from propargyl amines.

More recently, it was reported the use of an indenediide palladium complex as efficient catalyst for promoting this kind of transformation with a variety of differently substituted substrates (including propargyl amines bearing an internal triple bond), under mild conditions (0.5–1 bar of CO_2_, 40–80 °C in DMSO as the solvent), although with a higher catalyst loading (1–5 mol%) (Scheme 26) [103]. The *Z* configuration around the exocyclic double bond, observed for the 5-alkylideneoxazolidin-2-ones **26** obtained from propargyl amines with internal triple bond, was compatible with the *anti* 5-*exo*-*dig* cyclization pathway, as shown in Scheme 26. Detailed DFT investigations allowed identifying the cyclization step as the rate-determining step of the process.

**Scheme 27:**
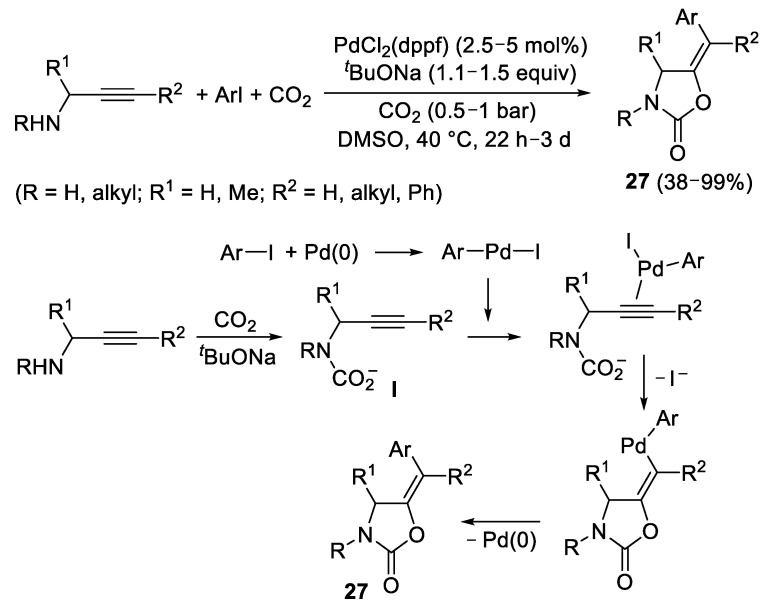
Synthesis of 5-arylideneoxazolidin-2-ones **27** from propargyl amines and aryl iodides.

Carboxylation of propargyl amines in the presence of aryl halides under the catalysis of Pd(0), with formation of 5-arylideneoxazolidin-2-ones **27**, is also possible, as shown in Scheme 27 [104]. The process starts with the oxidative addition of the aryl iodide to Pd(0) [formed in situ from PdCl_2_(dppf)] to give an Ar–Pd–I complex. On the other hand, a carbamate intermediate **I** is also formed by the reaction of the propargyl amine with CO_2_ in the presence of *t*-BuONa as base. Coordination of the triple bond of **I** to the Pd(II) center of the ArPdI species then takes place, followed by 5-*exo*-*dig* cyclization and reductive elimination to give the final product **27** with regeneration of Pd(0).

**Scheme 28:**
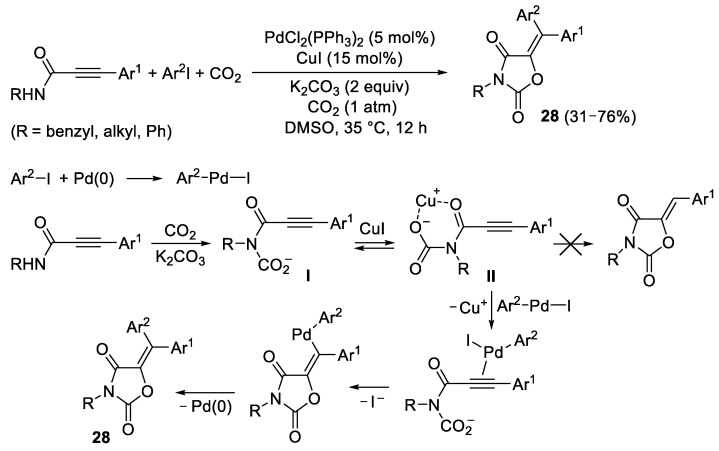
Synthesis of 5-arylideneoxazolidin-2,4-diones **28** from propargyl amides and aryl iodides.

In a similar manner, more recently 5-arylideneoxazolidine-2,4-diones **28** were synthesized starting from propargylic amides, aryl halides, and CO_2_, in the presence of PdCl_2_(PPh_3_)_2_ as the catalytic precursor, CuI as cocatalyst, and potassium carbonate as base (Scheme 28) [105]. The role of CuI was believed to be related to the possible stabilization of the carbamate intermediate **I** (by chelation of the amide carbonyl and the carboxylate group, leading to complex **II**), which avoids protonolysis, leading to oxazolidinones not incorporating the aryl moiety.

## 4. Palladium-Catalyzed Incorporation of Carbon Dioxide into Other Substrates

Under suitable conditions, aryl halides and triflates can undergo palladium-catalyzed carboxylation with formation of important compounds.

**Scheme 29:**
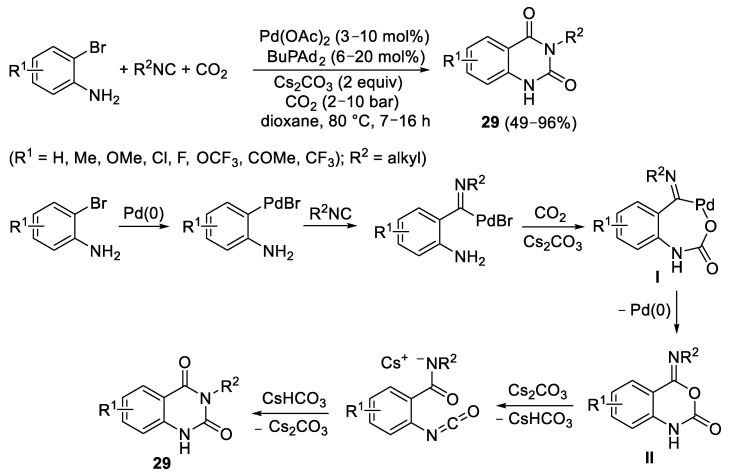
Synthesis of quinazoline-1,4(1*H*,3*H*)-diones **29** from 2-bromoanilines and isocyanides.

In 2017, the groups of Maes and Beller reported the Pd(0)-catalyzed reaction of 2-bromoanilines with CO_2_ and isocyanides to give quinazoline-1,4(1*H*,3*H*)-diones **29** [106]. Reactions were performed in the presence of Pd(OAc)_2_ as the catalyst precursor, in the presence of BuPdAd_2_ (Ad = adamantly) as ligand and Cs_2_CO_3_ as base, in dioxane as the solvent at 80 °C and under 10 bar of CO_2_ (Scheme 29). In the simplified version of the mechanism, oxidative addition of the Ar–Br bond to Pd(0) takes place, followed by insertion of the isocyanide. The reaction of the amino group with CO_2_ in the presence of the base then leads to a palladium carbamate intermediate **I**, which undergoes reductive elimination to yield Pd(0) and a 4-imino-1,4-dihydro-2*H*-benzo[*d*][1,3]oxazin-2-one intermediate **II**, from which the final product is obtained by base-promoted rearrangement (Scheme 29).

**Scheme 30:**
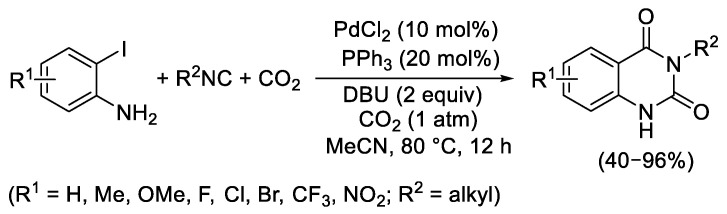
Synthesis of quinazoline-1,4(1*H*,3*H*)-diones from 2-iodoanilines and isocyanides.

**Scheme 31:**
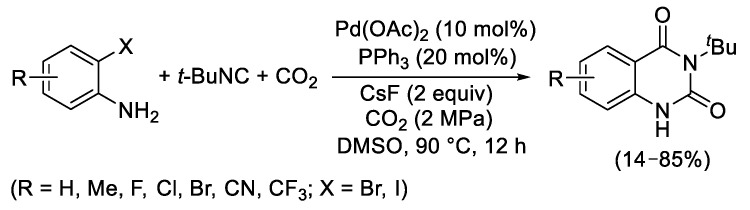
Synthesis of quinazoline-1,4(1*H*,3*H*)-diones from 2-haloanilines and *tert*-butyl isocyanide.

In the same year (2017), independently, the group of Wang and Ji published exactly the same transformation from 2-iodoanilines under atmospheric pressure of carbon dioxide, using PdCl_2_ in the presence of PPh_3_ as the catalyst precursor and DBU as the base, in MeCN at 80 °C (Scheme 30) [107]. Additionally, in 2018 Zhang and coworkers reported the use of both 2-bromo- and 2-iodoanilines using catalytic amounts of Pd(OAc)_2_ in the presence of PPh_3_ as ligand and CsF as base, under 2 MPa of CO_2_, in DMSO at 90 °C (Scheme 31) [108].

**Scheme 32:**
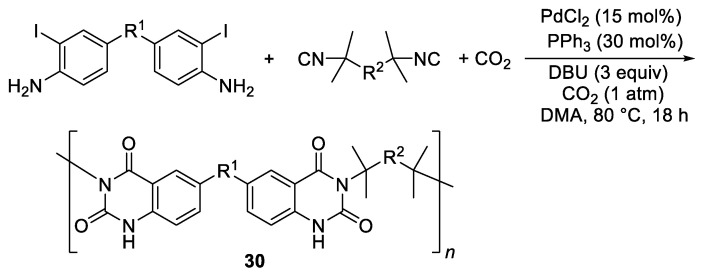
Synthesis of heterocyclic polymers **30** from bis(2-iodoaniline)s and diisocyanides.

Interestingly, very recently this kind of reactivity has been exploited for the synthesis of new heterocyclic polymers **30** with self-assembly and sensing properties, starting from bis(2-iodoaniline) and diisocyanide monomers, using PdCl_2_ and PPh_3_ as the catalyst precursor, under 1 atm of CO_2_, in DMA at 80 °C for 18 h (Scheme 32) [109].

**Scheme 33:**
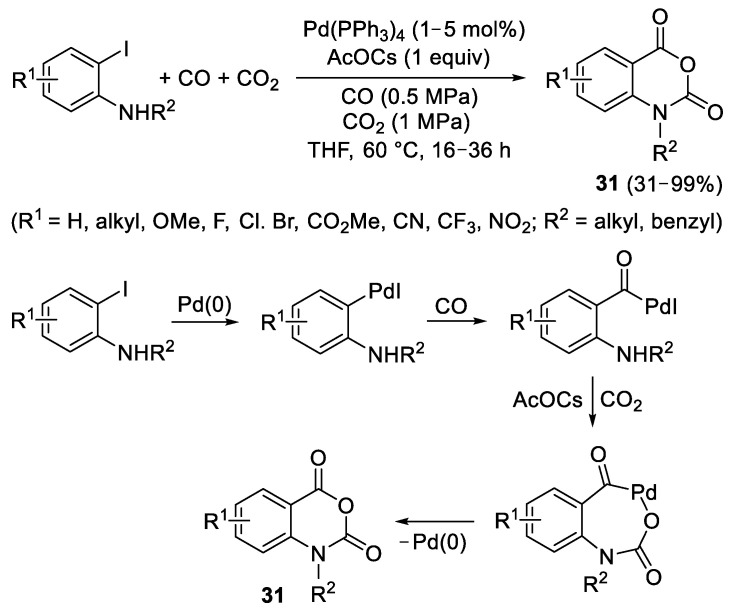
Synthesis isatoic anhydrides **31** from 2-iodoanilines.

Palladium-catalyzed incorporation of CO and CO_2_ into 2-iodoanilines to give isatoic anhydrides **31** has also been reported [110]. Reactions were carried out in THF at 60 °C under 1 MPa of CO_2_ and 0.5 MPa of CO, in the presence of Pd(PPh_3_)_4_ as catalyst and AcOCs as base (Scheme 33). Mechanistically, the process is similar to that seen in Scheme 29, with CO in place of the isocyanide (and without the final rearrangement step) (Scheme 33).

**Scheme 34:**
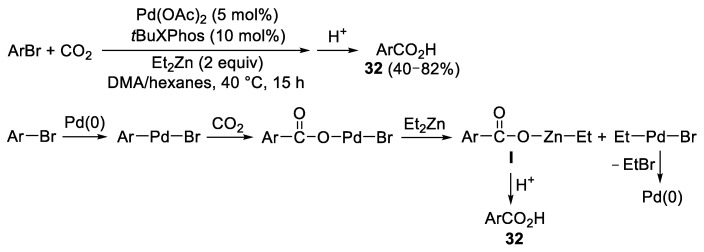
Synthesis of benzoic acids **32** from aryl bromides.

In 2009, Correa and Martín reported the first example of palladium-catalyzed carboxylation of aryl bromides to benzoic acids **32** [111]. Reactions were carried out with Pd(OAc)_2_ as the catalyst precursor in the presence of *t*BuXPhos as ligand (*t*BuXPhos = di-*tert*-butyl(2′,4′,6′-triisopropyl-[1,1′-biphenyl]-2-yl)phosphine) and 2 equiv of Et_2_Zn as reductant, in DMA/hexanes at 40 °C and under 10 atm of CO_2_ (Scheme 34). The proposed mechanism involves the oxidative addition of the aryl bromide to the in situ formed Pd(0), followed by carbon dioxide insertion and transmetallation with Et_2_Zn to give a zinc carboxylate **I** and an Et–Pd–Br species. Reductive elimination from the latter then regenerates Pd(0), while the benzoic acid product is obtained from zinc carboxylate following acidic work-up (Scheme 34).

**Scheme 35:**
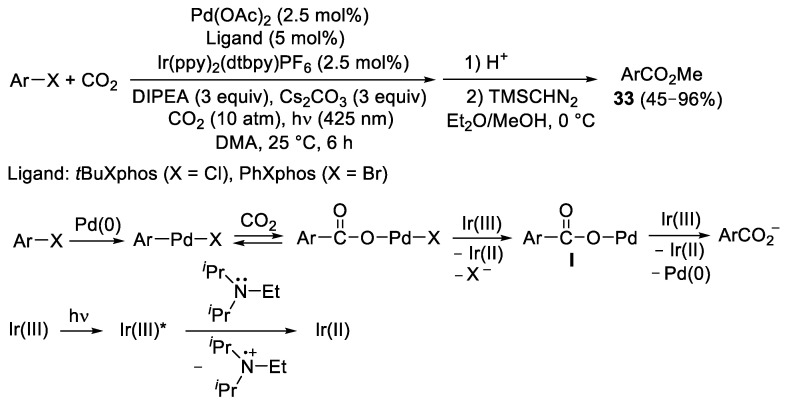
Synthesis of methyl benzoates **33** from aryl halides.

More recently, the first visible-light driven carboxylation of aryl halides (chlorides or bromides) to give methyl benzoates **33**, catalyzed by Pd(0) in conjunction with Ir(ppy)_2_(dtbpy)(PF_6_) as a photoredox catalyst (dtbpy = 4,4′-di-*tert*-butyl-2,2′-bipyridine), has been reported [112]. The optimized conditions involved the use of Pd(OAc)_2_ as the Pd(0) precursor, *t*-BuXPhos (with aryl chlorides) or PhXPhos (with aryl bromides; PhXPhos = 2-diphenylphosphino-2′,4′,6′-triisopropylbiphenyl) as ligand, in the presence of Cs_2_CO_3_ as base and DIPEA (DIPEA = *N*,*N*-diisopropylethylamine) as electron-donor species, in DMA as the solvent at r.t. and under 1 atm of CO_2_. The initially formed carboxylates were converted into the methyl esters by acidic quenching and subsequent reaction with TMSCHN_2_ (Scheme 35). This method avoids the use of metallic reductants (such as Et_2_Zn, seen before, Scheme 34) and is also compatible with aryl chlorides, unreactive under the conditions of Scheme 34. It is worth noting that the carboxylation of aryl halides with carbon dioxide can also be catalyzed by first-row transition metals, including copper [113], nickel [114], and cobalt [115] catalysts. However, only palladium catalysis seems to be compatible with visible light-promoted conditions so far. A possible mechanism starts with the oxidative addition of the aryl halide to Pd(0), followed by reversible CO_2_ insertion. A single-electron reduction by an Ir(II) complex then takes place, with formation of a Pd(I) carboxylate species **I** and an Ir(III) species. The Pd(I) carboxylate finally undergoes a second single-electron reduction to give the aryl carboxylate with regeneration of Pd(0). The Ir(III) species is reconverted into Ir(II) by photoexcitation followed by the reaction of the excited Ir(III)* species with DIPEA (Scheme 35).

**Scheme 36:**
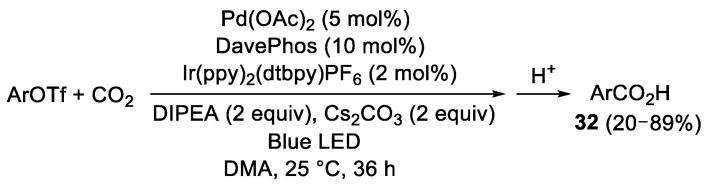
Synthesis of benzoic acids **32** from aryl triflates.

In a similar way, recently aryl triflates have been converted into benzoic acids **32** by Pd(0)-catalyzed, visible light-promoted carboxylation, carried out in the presence of Pd(OAc)_2_ as the catalyst precursor, DavePhos as ligand [DavePhos = 2-dicyclohexylphosphino-2′-(*N*,*N*-dimethylamino)biphenyl] and Ir(ppy)_2_(dtbpy)PF_6_ as photoredox cocatalyst (ppy = polypyrrole) [116]. Reactions were carried out under conditions similar to those seen above for aryl halides, namely, at r.t. and atmospheric pressure of CO_2_, in DMA as the solvent and in the presence of Cs_2_CO_3_ as base and DIPEA as electron donor (Scheme 36).

## 5. Conclusions

The versatility of palladium-based catalysts has been successfully exploited also in the efficient conversion of carbon dioxide into high value added organic molecules of applicative and pharmacological interest. Many different important Pd-catalyzed carboxylation processes have been developed and discussed in this review, mainly based on Pd(0) catalysis, although important Pd(II)-catalyzed reactions have also been reported. Particularly important results have been achieved in the Pd(0)-promoted CO_2_ incorporation into small rings (such as suitably functionalized epoxides and aziridines) as well as into suitably functionalized alkenes, allenes, or alkynes, to give highly important heterocyclic derivatives, such as cyclic carbonates, oxazolidinones, etc. Under Pd(II) catalysis, particularly important results have been achieved with propargyl amines as substrates, with formation of oxazolidinones, which could also incorporate an exocyclic estereal function working in the presence of CO together with CO_2_ under appropriate conditions.

On this grounds, it is expected that in the future palladium will play a major role in CO_2_ utilization and incorporation into suitable substrates, possibly in the presence of other promoting species (either metal-based or organo-based) as cocatalyst(s), which will allow us to achieve more demanding processes for the direct and selective synthesis of complex molecular architectures.

## Data Availability

Not applicable.

## References

[1] Park J.H., Yang J., Kim D., Gim H., Choi W.Y., Lee J.W. (2022). Review of recent technologies for transforming carbon dioxide to carbon materials. Chem. Eng. J..

[2] Al-Rowaili F.N., Zahid U., Onaizi S., Khaled M., Jamal A., AL-Mutairi E.M. (2021). A review for Metal-Organic Frameworks (MOFs) utilization in capture and conversion of carbon dioxide into valuable products. J. CO_2_ Util..

[3] Hussin F., Aroua M.K. (2021). Recent advances in low-temperature electrochemical conversion of carbon dioxide. Rev. Chem. Eng..

[4] Wang D., Xie Z., Porosoff M.D., Chen J.G. (2021). Recent advances in carbon dioxide hydrogenation to produce olefins and aromatics. Chem.

[5] Wickramasinghe S., Wang J., Morsi B., Li B. (2021). Carbon dioxide conversion to nanomaterials: Methods, applications, and challenges. Energy Fuels.

[6] Zhang T., Zhong J., Wu Z. (2021). Recent advances in catalytic conversion of carbon dioxide to propiolic acids over coinage-metal-based catalysts. J. Energy Chem..

[7] Sable D.A., Vadagaonkar K.S., Kapdi A.R., Bhanage B.M. (2021). Carbon dioxide based methodologies for the synthesis of fine chemicals. Org. Biomol. Chem..

[8] Guo S., Asset T., Atanassov P. (2021). Catalytic hybrid electrocatalytic/biocatalytic cascades for carbon dioxide reduction and valorization. ACS Catal..

[9] Chowdhury I.H., Chowdhury A.H., Sarkar P., Islam S.M. (2021). Chemical fixation of carbon dioxide by heterogeneous porous catalysts. ChemNanoMat.

[10] Pires Da Mata Costa L., Micheline Vaz De Miranda D., Couto De Oliveira A.C., Falcon L., Stella Silva Pimenta M., Guilherme Bessa I., Juarez Wouters S., Andrade M.H.S., Pinto J.C. (2021). Capture and reuse of carbon dioxide (CO_2_) for a plastics circular economy: A review. Processes.

[11] Garba M.D., Usman M., Khan S., Shehzad F., Galadima A., Ehsan M.F., Ghanem A.S., Humayun M. (2021). CO_2_ towards fuels: A review of catalytic conversion of carbon dioxide to hydrocarbons. J. Environ. Chem. Eng..

[12] Calmanti R., Selva M., Perosa A. (2021). Tandem catalysis: One-pot synthesis of cyclic organic carbonates from olefins and carbon dioxide. Green Chem..

[13] Zhang X., Zhang G., Song C., Guo X. (2021). Catalytic conversion of carbon dioxide to methanol: Current status and future perspective. Front. Energy Res..

[14] Manaka Y. (2021). Carbon dioxide utilization by using organic or metal catalysts. J. Jpn. Pet. Inst..

[15] Gao W., Liang S., Wang R., Jiang Q., Zhang Y., Zheng Q., Xie B., Toe C.Y., Zhu X., Wang J. (2020). Industrial carbon dioxide capture and utilization: State of the art and future challenges. Chem. Soc. Rev..

[16] Shi Y., Pan B.-W., Zhou Y., Zhou J., Liu Y.-L., Zhou F. (2020). Catalytic enantioselective synthesis using carbon dioxide as a C1 synthon. Org. Biomol. Chem..

[17] Truong C.C., Mishra D.K. (2020). Catalyst-free fixation of carbon dioxide into value-added chemicals: A review. Environ. Chem. Lett..

[18] Truong C.C., Mishra D.K. (2020). Recent advances in the catalytic fixation of carbon dioxide to value-added chemicals over alkali metal salts. J. CO_2_ Util..

[19] Fan Z., Zhang Z., Xi C. (2020). Light-mediated carboxylation using carbon dioxide. ChemSusChem.

[20] Dibenedetto A., Nocito F. (2020). The future of carbon dioxide chemistry. ChemSusChem.

[21] Zhou C., Li M., Yu J., Sun S., Cheng J. (2020). Recent progress in the carboxylation/cyclization reactions using carbon dioxide as the C1 source. Chin. J. Org. Chem..

[22] Zhang Z., Pan S.-Y., Hao L., Cai J., Olabi A.G., Anthony E.J., Manovic V. (2020). Recent advances in carbon dioxide utilization. Renew. Sust. Energ. Rev..

[23] Li M., Abdolmohammadi S., Hoseininezhad-Namin M.S., Behmagham F., Vessally E. (2020). Carboxylative cyclization of propargylic alcohols with carbon dioxide: A facile and green route to alpha-methylene cyclic carbonates. J. CO_2_ Util..

[24] Huang J., Worch J.C., Dove A.P., Coulembier O. (2020). Update and challenges in carbon dioxide-based polycarbonate synthesis. ChemSusChem.

[25] Fu Z., Yang Q., Liu Z., Chen F., Yao F., Xie T., Zhong Y., Wang D., Li J., Li X. (2019). Photocatalytic conversion of carbon dioxide: From products to design the catalysts. J. CO_2_ Util..

[26] Gomez J.E., Kleij A.W. (2019). Catalytic nonreductive valorization of carbon dioxide into fine chemicals. Adv. Organomet. Chem..

[27] Dalpozzo R., Della Ca’ N., Gabriele B., Mancuso R. (2019). Recent advances in the chemical fixation of carbon dioxide: A green route to carbonylated heterocycle synthesis. Catalysts.

[28] Wu J., Sharifi T., Gao Y., Zhang T., Ajayan P.M. (2019). Emerging carbon-based heterogeneous catalysts for electrochemical reduction of carbon dioxide into value-added chemicals. Adv. Mater..

[29] Shukia S.K., Khokarale S.G., Bui T.Q., Mikkola J.P.T. (2019). Ionic liquids: Potential materials for carbon dioxide capture and utilization. Front. Mater..

[30] Dabral S., Schaub T. (2019). The use of carbon dioxide (CO_2_) as a building block in organic synthesis from an industrial perspective. Adv. Synth. Catal..

[31] Seo H., Nguyen L.V., Jamison T.F. (2019). Using carbon dioxide as a building block in continuous flow synthesis. Adv. Synth. Catal..

[32] Hong J., Li M., Zhang J., Sun B., Mo F. (2019). C-H bond carboxylation with carbon dioxide. ChemSusChem.

[33] Li J.-Y., Song Q.-W., Zhang K., Liu P. (2019). Catalytic conversion of carbon dioxide through C-N bond formation. Molecules.

[34] Liang J., Huang Y.-B., Cao R. (2019). Metal-organic frameworks and porous organic polymers for sustainable fixation of carbon dioxide into cyclic carbonates. Coord. Chem. Rev..

[35] Fujinami T., Suzuki T., Kamiya M., Fukuzawa S.-I., Sakai S. (1985). Palladium catalyzed reaction of butadiene monoxide with carbon dioxide. Chem. Lett..

[36] Trost B.M., Angle S.M. (1985). Palladium-mediated vicinal cleavage of allyl epoxides with retention of stereochemistry: A cis hydroxylation equivalent. J. Am. Chem. Soc..

[37] Trost B.M., Lynch J.K., Angle S.M. (1987). Asymmetric cis-hydroxylation via epoxidation-carboxylation: A formal synthesis of (+)-Citreoviral. Tetrahedron Lett..

[38] Wershofen S., Scharf H.-D. (1988). Synthesis of side-chain unsaturated endo- and exo-brevicomis. Representative of pheromone analogs in the dioxabicyclo [3.2.1] octane series. Synthesis.

[39] Fontana F., Chen C.C., Aggarwal V.K. (2011). Palladium-catalyzed insertion of CO_2_ into vinylaziridines: New route to 5-vinyloxazolidinones. Org. Lett..

[40] Chen K., Jiang M., Zhang Z., Wei Y., Shi M. (2011). Palladium (0)-catalyzed reaction of cyclopropylidenecycloalkanes with carbon dioxide. Eur. J. Org. Chem..

[41] Greco G.E., Gleason B.L., Lowery T.A., Kier M.J., Hollander L.B., Gibbs S.A., Worthy A.D. (2007). Palladium-catalyzed [3+2] cycloaddition of carbon dioxide and trimethylenemethane under mild conditions. Org. Lett..

[42] Yoshida M., Ohsawa Y., Ihara M. (2004). Palladium-catalyzed carbon dioxide elimination-fixation reaction of 4-methoxycarbonyloxy-2-buten-1-ols. J. Org. Chem..

[43] Yoshida M., Ohsawa Y., Ihara M. (2006). Palladium-catalyzed carbon dioxide elimination–fixation reaction of 6-methoxycarbonyloxy-2,4-hexadien-1-ols. Tetrahedron.

[44] Sun L., Ye J.-H., Zhou W.-J., Zeng X., Yu D.-G. (2018). Oxy-alkylation of allylamines with unactivated alkyl bromides and CO_2_ via visible-light-driven palladium catalysis. Org. Lett..

[45] Sun S., Zhou C., Yu J.-T., Cheng J. (2019). Visible-light-driven palladium-catalyzed oxy-alkylation of 2-(1-arylvinyl)anilines by unactivated alkyl bromides and CO_2_: Multicomponent reactions toward 1,4-dihydro-2*H*-3,1-benzoxazin-2-ones. Org. Lett..

[46] Tsuda T., Yamamoto T., Saegusa T. (1992). Palladium-catalyzed cycloaddition of carbon dioxide with methoxyallene. J. Organomet. Chem..

[47] Xiong W., Yan D., Qi C., Jiang H. (2018). Palladium-catalyzed four component cascade reaction for the synthesis of highly functionalized acyclic *O*,*O*-acetals. Org. Lett..

[48] Xiong W., Cheng R., Wu B., Wu W., Qi C., Jiang H. (2020). Palladium-catalyzed regioselective cascade reaction of carbon dioxide, amines and allenes for the synthesis of functionalized carbamates. Sci. China Chem..

[49] Higuchi Y., Mita T., Sato Y. (2017). Palladium-catalyzed intramolecular arylative carboxylation of allenes with CO_2_ for the construction of 3-substituted indole-2-carboxylic acids. Org. Lett..

[50] Kayaki Y., Mori N., Ikayara T. (2009). Palladium-catalyzed carboxylative cyclization of α-allenyl amines in dense carbon dioxide. Tetrahedron Lett..

[51] Uemura K., Shiraishi D., Noziri M., Inoue Y. (1999). Preparation of cyclic carbonates from alkadienols, CO_2_, and aryl or vinylic halides catalyzed by a palladium complex. Bull. Chem. Soc. Jpn..

[52] Li S., Ye J., Yuan W., Ma S. (2013). Highly regioselective three-component palladium-catalyzed synthesis of 5-vinyloxazolidin-2-ones from 2,3-allenyl amines, organic iodides, and carbon dioxide. Tetrahedron.

[53] Ye J., Li S., Ma S. (2013). Gorlos-Phos: Addressing the stereoselectivity in palladium-catalyzed exo-mode cyclization of allenes with a nucleophilic functionality. Org. Biomol. Chem..

[54] Sasaki Y., Inoue Y., Hashimoto H. (1976). Reaction of carbon dioxide with butadiene catalysed by palladium complexes. Synthesis of 2-ethylidenehept-5-en-4-olide. J. Chem. Soc. Chem. Commun..

[55] Inoue Y., Sasaki Y., Hashimoto H. (1978). Incorporation of CO_2_ in butadiene dimerization catalyzed by palladium complexes. Formation of 2-ethylidene-5-hepten-4-olide. Bull Chem. Soc. Jpn..

[56] Musco A., Perego C., Tartiari V. (1978). Telomerization reactions of butadiene and CO_2_ catalyzed by phosphine Pd (0) complexes: (E)-2-ethylidenehept-6-en-5-olide and octadienyl esters of 2-ethylidenehepta-4, 6-dienoic acid. Inorg. Chim. Acta.

[57] Musco A. (1980). Co-oligomerization of butadiene and carbon dioxide catalysed by tertiary phosphine-palladium complexes. J. Chem. Soc. Perkin Trans. 1.

[58] Behr A., Juszak K.-D., Keim W. (1983). Synthese von 2-ethylide-6-hepten-olid. Synthesis.

[59] Behr A., Juszak K.-D. (1983). Palladium-catalyzed reaction of butadiene and carbon dioxide. J. Organomet. Chem..

[60] Behr A., Henze G. (2011). Use of carbon dioxide in chemical syntheses via a lactone intermediate. Green Chem..

[61] Ferretti F., Sharif M., Dastgir S., Ragaini F., Jackstell R., Beller M. (2017). Selective palladium-catalysed synthesis of diesters: Alkoxycarbonylation of a CO_2_-butadiene derived *δ*-lactone. Green Chem..

[62] Chen L., Li Y., Yue S., Ling J., Ni X., Shen Z. (2017). Chemoselective RAFT Polymerization of a trivinyl monomer derived from carbon dioxide and 1, 3-butadiene: From linear to hyperbranched. Macromolecules.

[63] Moon S., Masada K., Nozaki K. (2019). Reversible polymer-chain modification: Ring-opening and closing of polylactone. J. Am. Chem. Soc..

[64] Nakano R., Ito S., Nozaki K. (2014). Copolymerization of carbon dioxide and butadiene via a lactone intermediate. Nat. Chem..

[65] Dinjus E., Leitner W. (1995). New insights into the palladium-catalysed synthesis of δ-lactones from 1, 3-dienes and carbon dioxide. Appl. Organomet. Chem..

[66] Behr A., He R., Juszak K.-D., Krüger C., Tsay Y.-H. (1986). Steuerungsmöglichkeiten bei der übergangsmetall-katalysierten Umsetzung von 1, 3-Dienen mit Kohlendioxid. Chem. Ber..

[67] Behr A. (1988). Carbon dioxide as an alternative C1 synthetic unit: Activation by transition-metal complexes. Angew. Chem. Int. Ed..

[68] Braunstein P., Matt D., Nobel D. (1988). Carbon dioxide activation and catalytic lactone synthesis by telomerization of butadiene and carbon dioxide. J. Am. Chem. Soc..

[69] Braunstein P., Matt D., Nobel D. (1988). Reactions of carbon dioxide with carbon-carbon bond formation catalyzed by transition-metal complexes. Chem. Rev..

[70] Elsagir A.R., Gaßner F., Görls H., Dinjus E. (2000). Bidentate ferrocenylphosphines and their palladium(II)dichloride complexes—X-ray structural and NMR spectroscopic investigations and first results of their characteristics in the Pd-catalysed cooligomerisation of 1, 3-butadiene with CO_2_. J. Organomet. Chem..

[71] Behr A., Becker M. (2006). The telomerisation of 1, 3-butadiene and carbon dioxide: Process development and optimisation in a continuous miniplant. Dalton Trans..

[72] Dai Y., Feng X., Wang B., He R., Bao M. (2012). Preparation and application of air-stable *P*, *N*-bidentate ligands for the selective synthesis of δ-lactone via the palladium-catalyzed telomerization of 1, 3-butadiene with carbon dioxide. J. Organomet. Chem..

[73] Song J., Feng X., Yamamoto Y., Almansour A.I., Arumugam N., Kumar R.S., Bao M. (2016). Selective synthesis of δ-lactone via palladium nanoparticles-catalyzed telomerization of CO_2_ with 1, 3-butadiene. Tetrahedron Lett..

[74] Sharif M., Jackstell R., Dastgir S., Al-Shihi B., Beller M. (2017). Efficient and selective palladium-catalyzed telomerization of 1,3-butadiene with carbon dioxide. ChemCatChem.

[75] Balbino J.M., Dupont J., Bayón J.C. (2018). Telomerization of 1, 3-butadiene with carbon dioxide: A highly efficient process for δ-lactone generation. ChemCatChem.

[76] Iratani K., Yanagihara N., Utimoto K. (1986). Carboxylative coupling of propargylic alcohols with allyl chloride. J. Org. Chem..

[77] Inoue Y., Itoh Y., Yen I.-F., Imaizumi S. (1990). Palladium (0)-catalyzed carboxylative cyclized coupling of propargylic alcohol with aryl halides. J. Mol. Catal..

[78] Sun S., Wang B., Gu N., Yu J.-T., Cheng J. (2017). Palladium-catalyzed arylcarboxylation of propargylic alcohols with CO_2_ and aryl halides: Access to functionalized α-alkylidene cyclic carbonates. Org. Lett..

[79] Song B., Bai T., Xu X., Chen X., Liu D., Guo J., Qin A., Ling J., Tang B.Z. (2019). Multifunctional linear and hyperbranched five-membered cyclic carbonate-based polymers directly generated from CO_2_ and alkyne-based three-component polymerization. Macromolecules.

[80] Yoshida M., Ihara M. (2004). Novel methodologies for the synthesis of cyclic carbonates. Chem. Eur. J..

[81] Yoshida M., Ihara M. (2001). Palladium-catalyzed domino reaction of 4-methoxycarbonyloxy-2-butyn-1-ols with phenols: A novel synthetic method for cyclic carbonates with recycling of CO_2_. Angew. Chem. Int. Ed..

[82] Yoshida M., Fujita M., Ishii T., Ihara M. (2003). A novel methodology for the synthesis of cyclic carbonates based on the palladium-catalyzed cascade reaction of 4-methoxycarbonyloxy-2-butyn-1-ols with phenols, involving a novel carbon dioxide elimination-fixation process. J. Am. Chem. Soc..

[83] Yoshida M., Fujita M., Ihara M. (2003). Palladium-catalyzed enantiospecific reaction of propargylic carbonates with phenols: Cascade chirality transfer. Org. Lett..

[84] Yoshida M., Murao T., Sugimoto K., Ihara M. (2007). Palladium-catalyzed three-component coupling of propargylic oxiranes, phenols and carbon dioxide. Synlett.

[85] Bacchi A., Chiusoli G.P., Costa M., Gabriele B., Righi C., Salerno G. (1997). Palladium-catalysed sequential carboxylation–alkoxycarbonylation of acetylenic amines. Chem. Commun..

[86] Chiusoli G.P., Costa M., Gabriele B., Salerno G. (1999). Sequential reaction of carbon dioxide and carbon monoxide with acetylenic amines in the presence of a palladium catalyst. J. Mol. Catal. A Chem..

[87] Gabriele B. (2021). Carbon Monoxide in Organic Synthesis–Carbonylation Chemistry.

[88] Zhu C., Liu J., Li M.B., Bäckvall J.E. (2020). Palladium-catalyzed oxidative dehydrogenative carbonylation reactions using carbon monoxide and mechanistic overviews. Acc. Chem. Res..

[89] Mancuso R., Della Ca N., Veltri L., Ziccarelli I., Gabriele B. (2019). PdI_2_-based catalysis for carbonylation reactions: A personal account. Catalysts.

[90] Peng J.B., Geng H.-Q., Wu X.-F. (2019). The chemistry of CO: Carbonylation. Chem.

[91] Gabriele B. (2018). Recent advences in the PdI_2_-catalyzed carbonylative synthesis of heterocycles from acetylenic substrates: A personal account. Targets Heterocycl Syst..

[92] Gabriele B., Solé D., Fernández I. (2018). Synthesis of heterocycles by palladium-catalyzed carbonylative reactions. Advances in Transition-Metal Mediated Heterocyclic Synthesis.

[93] Wu X.-F., Neumann H., Beller N. (2013). Palladium-catalyzed oxidative carbonylation reactions. ChemSusChem.

[94] Wu X.-F., Neumann H., Beller N. (2013). Synthesis of heterocycles via palladium-catalyzed carbonylations. Chem. Rev..

[95] Gabriele B., Mancuso R., Salerno G. (2012). Oxidative carbonylation as a powerful tool for the direct synthesis of carbonylated heterocycles. Eur. J. Org. Chem..

[96] Liu Q., Zhang H., Lei A. (2011). Oxidative carbonylation reactions: Organometallic compounds (R-M) or hydrocarbons (R-H) as nucleophiles. Angew. Chem. Int. Ed..

[97] Gabriele B., Salerno G., Costa M. (2006). Oxidative Carbonylations. Top. Organomet. Chem..

[98] Gabriele B., Salerno G., Costa M., Chiusoli G.P. (2004). Recent advances in the synthesis of carbonyl compounds by palladium- catalyzed oxidative carbonylation reactions of unsaturated substrates. Curr. Org. Chem..

[99] Gabriele B., Salerno G., Costa M., Chiusoli G.P. (2003). Recent developments in the synthesis of heterocyclic derivatives by PdI_2_-catalyzed oxidative carbonylation reactions. J. Organomet. Chem..

[100] Gabriele B., Costa M., Salerno G., Chiusoli G.P. (1994). An efficient and selective palladium-catalysed oxidative dicarbonylation of alkynes to alkyl- or aryl-maleic esters. J. Chem. Soc. Perkin Trans. 1.

[101] Gabriele B., Salerno G., Crich D. (2006). PdI2. e-EROS (Electronic Encyclopedia of Reagents for Organic Synthesis).

[102] Shi M., Shen Y.-M. (2002). Transition-metal catalyzed reactions of propargylamine with carbon dioxide and carbon disulfide. J. Org. Chem..

[103] Brunel P., Monot J., Kefalidis C.E., Maron L., Martin-Vaca B., Bourissou D. (2017). Valorization of CO_2_: Preparation of 2-oxazolidinones by metal-ligand cooperative catalysis with SCS indenediide Pd complexes. ACS Catal..

[104] García-Dominguez P., Fehr L., Rusconi G., Nevado C. (2016). Palladium-catalyzed incorporation of atmospheric CO_2_: Efficient synthesis of functionalized oxazolidinones. Chem. Sci..

[105] Zhou C., Dong Y., Yu J.-T., Sun S., Cheng J. (2019). Palladium/copper-catalyzed multicomponent reactions of propargylic amides, halohydrocarbons and CO_2_ toward functionalized oxazolidine-2, 4-diones. Chem. Commun..

[106] Mampuys P., Neumann H., Sergeyev S., Orru R.V.A., Jiao H., Spannenberg A., Maes B.U.W., Beller M. (2017). Combining isocyanides with carbon dioxide in palladium-catalyzed heterocycle synthesis: *N*3-substituted quinazoline-2, 4 (1*H*,3*H*)-diones via a three-component reaction. ACS Catal..

[107] Xu P., Wang F., Wei T.-Q., Yin L., Wang S.-Y., Ji S.-J. (2017). Palladium-catalyzed incorporation of two C1 building blocks: The reaction of atmospheric CO_2_ and isocyanides with 2-iodoanilines leading to the synthesis of quinazoline-2, 4 (1*H*,3*H*)-diones. Org. Lett..

[108] Zhang W.-Z., Li H., Zeng Y., Tao X., Lu X. (2018). Palladium-catalyzed cyclization reaction of *o*-haloanilines, CO_2_ and isocyanides: Access to quinazoline-2, 4-(1*H*,3*H*)-diones. Chin. J. Chem..

[109] Liu D., Song B., Wang J., Li B., Wang B., Li M., Qin A., Tang B.Z. (2021). CO_2_-Involved and isocyanide-based three-component polymerization toward functional heterocyclic polymers with self-assembly and sensing properties. Macromolecules.

[110] Zhang W.-Z., Zhang N., Sun Y.-Q., Ding Y.-W., Lu X.-B. (2017). Palladium-catalyzed cyclization reaction of *o*-iodoanilines, CO_2_, and CO: Access to isatoic anhydrides. ACS Catal..

[111] Correa A., Martín R. (2009). Palladium-catalyzed direct carboxylation of aryl bromides with carbon dioxide. J. Am. Chem. Soc..

[112] Shimomaki K., Murata K., Martín R., Iwasawa N. (2017). Visible-light-driven carboxylation of aryl halides by the combined use of palladium and photoredox catalysts. J. Am. Chem. Soc..

[113] Tran-Vu H., Daugulis O. (2013). Copper-catalyzed carboxylation of aryl iodides with carbon dioxide. ACS Catal..

[114] Fujihara T., Nogi K., Xu T., Terao J., Tsuji Y. (2012). Nickel-catalyzed carboxylation of aryl and vinyl chlorides employing carbon dioxide. J. Am. Chem. Soc..

[115] Nogi K., Fujihara T., Terao J., Tsuji Y. (2015). Cobalt- and nickel-catalyzed carboxylation of alkenyl and sterically hindered aryl triflates utilizing CO_2_. J. Org. Chem..

[116] Bhunia S.K., Das P., Nandi S., Jana R. (2019). Carboxylation of aryl triflates with CO_2_ merging palladium and visible-light-protoredox catalysts. Org. Lett..

